# Single‐Step Genomic Predictions for Growth and Carcass Traits in Nordic Charolais and Hereford Cattle

**DOI:** 10.1111/jbg.70018

**Published:** 2025-10-02

**Authors:** Anahit Nazari‐Ghadikolaei, Freddy Fikse, Susanne Eriksson

**Affiliations:** ^1^ Department of Animal Biosciences Swedish University of Agricultural Sciences Uppsala Sweden; ^2^ Växa Uppsala Sweden

## Abstract

In order to investigate the applicability and efficiency of genomic selection for growth and carcass traits in Nordic beef cattle, single‐step genomic BLUP (ssGBLUP) was applied in 4321 Charolais and 4532 Hereford animals with information on approximately 43,000 SNPs each. Statistics including dispersion value (*b*1), accuracy ratio and the relative accuracy improvement were estimated for genotyped female animals in the validation set. For estimating dispersion, accuracy ratio and relative accuracy improvement, the Legarra–Reverter linear regression (LR) method was used by truncating the phenotypes after 2018, and the validation set comprised females born from 2019 to 2021. Moreover, for ssGBLUP, different alpha values of 0.95 and 0.70 were utilised as weights on the genomic information when the H matrix was blended for the genomic relationship matrix G and the pedigree relationship matrix A. In general, implementing ssGBLUP led to higher accuracy ratios and improved dispersion values (*b*1 value closer to the optimum value of one), compared to when using pedigree‐based BLUP (PBLUP). Using an alpha value of 0.70 gave a dispersion value closer to one compared with when using an alpha value of 0.95. Additionally, the relative accuracy estimation was improved substantially for several traits by using ssGBLUP instead of PBLUP, with the highest (30%) relative improvement for carcass conformation in Swedish Hereford cattle. In conclusion, ssGBLUP would be beneficial to implement in the future Nordic beef cattle breeding programs.

## 1 Background/Introduction

The importance of genetic improvement of beef breeds for Nordic beef production has increased as the number of dairy cows has decreased, and the use of beef semen in dairy herds is expected to further increase in the coming years (Clasen et al. 2021). Concerns have been raised about the climate impact of beef production, thereby highlighting the importance of increased efficiency also for other than the obvious economic reasons (Terry et al. 2021). The rate of genetic improvement of the animal material could be enhanced by utilising genomic information and selecting animals on genomic enhanced breeding values (GEBVs).

Genomic selection has been advantageous in animal breeding schemes by accelerating the genetic gain through improved accuracy of estimated breeding values (EBVs) for young animals, as well as shortened generation intervals (Guinan et al. 2023). Although multi‐step genomic prediction has been used in dairy cattle breeding programs, the method is less well suited for beef cattle due to smaller reference populations, limited use of artificial insemination, fewer progeny‐tested bulls and possibly a lower degree of relationship between animals in reference and validation population sets (Meuwissen et al. 2016). The more recently developed single‐step methodology for genomic prediction has been successfully introduced in different beef breeds, and one example is the application in Angus beef cattle in the United States (Lourenco et al. 2015).

Single‐step genomic best linear unbiased prediction (ssGBLUP) uses pedigree, phenotype and genotype information at the same time by combining the pedigree and genomic relationship matrices in an H matrix, which gives less bias compared to the multi‐step GBLUP (Misztal et al. 2009; Legarra et al. 2014). With ssGBLUP, non‐genotyped animals will benefit from the genomic information from their genotyped relatives by using the H matrix (Misztal et al. 2009).

A joint pedigree‐based BLUP evaluation of beef breeds in Sweden, Finland and Denmark was established in 2021 (NAV 2024). The purebred beef populations within each of these countries are relatively small, and an across‐country reference population for genomic prediction would be an attractive option. Clear benefits in terms of improved accuracy of breeding values of moving to international compared with national single‐step SNPBLUP evaluations have previously been shown for weaning weight in Limousine (Bonifazi et al. 2022). The level of genetic relationships between animals in the different populations is then important to consider, as it influences the value of including foreign animals in the reference population (Saatchi et al. 2013). In 2020, the use of SNP genotyping for parentage verification of Swedish and Finnish beef cattle was established. In addition, thousands of Nordic beef breed animals have been genotyped within the framework of recent research projects (Hietala et al. 2022). Taken together, these provide an opportunity for introducing genomic enhanced breeding values for beef cattle on a Nordic scale.

The aim of this study was therefore to investigate the feasibility and performance of within‐breed single‐step genomic prediction in Swedish, Finnish and Danish Charolais and Hereford animals, by considering genomic relatedness within and across countries, and estimating dispersion (*b*1) values, accuracy ratios and relative accuracy improvement.

## 2 Material and Methods

### 2.1 Animals, Pedigree and Phenotypes

Pedigree, growth and carcass trait data for Hereford and Charolais animals were provided from the Nordic Cattle Genetic Evaluation (NAV, Aarhus, Denmark) of beef breeds (Rius‐Vilarrasa et al. 2022). The observations were pre‐edited and pre‐adjusted for heterogeneous variance within country‐year‐breed‐sex class using a simple phenotypic adjustment by subtracting within‐class mean and then dividing by within‐class standard deviation, before multiplying by an overall standard deviation and adding back the within‐group mean, according to the routines for the NAV beef evaluation (Rius‐Vilarrasa et al. 2022). The pedigree files contained in total 417,886 Charolais and 507,135 Hereford animals, respectively. The growth and carcass traits included were birth weight (BW, kg), weaning weight gain (WWG, kg), post weaning gain (PWG, kg, only for Finnish and Swedish animals), yearling weight (YW, kg, only for Danish animals), slaughter daily gain (SDG, g/d), EUROP conformation class (SCONF, points 1–15) and EUROP fat class (SFAT, points 1–5). In total, the phenotype data included 358,198 Charolais and 406,857 Hereford animals with at least one trait record, whereof 291,883 Charolais and 267,238 Hereford animals had records on birth weight, which was the trait with the most complete recording (Table 1).

**TABLE 1:** Summary statistics for growth and carcass traits for Charolais and Hereford animals.

Trait	*N*	Min	Max	Mean	SD	*N* genotyped with records
Charolais						
BW	291,883	22.0	70.0	46.0	5.7	4126
WWG	175,578	66.6	413.3	294.0	46.3	3646
YW	13,105	178.5	787.7	491.2	87.4	44
PWG	117,547	−15.3	445.9	307.0	70.1	3316
SDG	142,003	61.6	1209.2	660.5	164.0	381
SCONF	134,671	0.6	16.6	9.6	2.05	379
SFAT	134,675	−0.5	5.7	2.3	0.8	379
Hereford						
BW	267,238	15.0	64.0	40.0	5.8	4168
WWG	152,155	32.0	408.5	211.9	46.9	3898
YW	21,939	117.8	769	440.8	93.1	237
PWG	88,523	−42.2	378.8	166.7	61.7	3177
SDG	198,307	−12.5	1099.8	533.0	147.1	405
SCONF	173,181	−2.0	16.6	7.1	1.9	401
SFAT	173,195	−0.3	5.2	3.0	0.7	401

*Note:* Due to adjustment for heterogeneous variances, negative minimum values and maximum values outside the point range occur.

Abbreviations: BW, birth weight (kg); PWG, post‐weaning gain (kg); SCONF, carcass conformation (points 1–15); SDG, slaughter daily gain (g); SFAT, carcass fatness (points 1–5); WWG, weaning weight gain (kg); YW, yearling weight (kg).

### 2.2 Genotyping and Quality‐Control

Genotype data for 9227 animals (4452 Charolais and 4775 Hereford) were obtained from NAV's database (NAV 2024). The majority of animals were genotyped by Eurofins in Denmark using recent versions of the EuroGenomics bovine medium‐dense SNP array containing close to 70 K SNPs. However, some (older) animals (*N* = 436) were genotyped on low‐density SNP arrays, and these were excluded from the study. The genome build UMD3.1 was used as the reference map for the combined SNP data. Quality control (QC) was performed for each breed separately using Plink v.1.7 (Purcell et al. 2007). For the quality control, we first removed SNPs with a call rate below 95%, minor allele frequency (MAF) of < 0.05 and Hardy–Weinberg equilibrium (HWE) exact test *p*‐value of < 1e‐6. Thereafter, animals with a call rate of less than 90% were deleted. Moreover, we removed 131 Charolais and 243 Hereford genotypes because their identities were either not found in the pedigree or they were identified to be duplicates. After QC, 4321 Charolais animals with autosomal 43,141 SNPs and 4532 Hereford animals with 40,988 autosomal SNPs remained for further analyses. The genotyped animals were born from 1999 to 2021, and 73% were females.

The number of genotyped animals from Denmark was very low compared to that from Sweden and Finland (Table 2). More than 80% of the genotyped animals had their own records for at least three of the traits in the data. Genotyped sires had 1–274 offspring with trait records, with an average of 31 offspring for Charolais bulls and 21 offspring for Hereford bulls (Table 3).

**TABLE 2:** Number of genotyped male and female Charolais and Hereford animals in the three Nordic countries Sweden (SWE), Finland (FIN) and Denmark (DNK).

Country	Charolais			Hereford		
	*N* males	*N* females	Birth years	*N* males	*N* females	Birth years
SWE	734	1585	2007–2021	473	1293	1999–2021
FIN	341	1383	2003–2021	470	1597	2006–2021
DNK	65	58	1999–2021	225	180	2005–2021

**TABLE 3:** Number of genotyped sires and dams and range of number of offspring (N_OS) per genotyped sire and dam from Sweden (SWE), Finland (FIN) and Denmark (DNK).

Trait	SWE sires		FIN sires		DNK sires		SWE dams		FIN dams		DNK dams	
	*N*	Range N_OS	*N*	Range N_OS	*N*	Range N_OS	*N*	Range N_OS	*N*	Range N_OS	*N*	Range N_OS
Charolais												
BW	293	1–341	56	1–146	38	1–254	1399	1–14	1073	1–16	59	1–13
WW	266	1–274	45	1–125	12	1–81	1291	1–13	967	1–15	28	1–10
PWG/YW^a^	240	1–223	28	1–42	12	1–79	1140	1–12	681	1–12	28	1–10
SDG	211	1–104	38	1–87	20	1–222	647	1–7	723	1–12	36	1–6
SCONF	211	1–104	38	1–87	20	1–195	647	1–7	723	1–12	30	1–6
SFAT	211	1–104	38	1–87	20	1–195	647	1–7	723	1–12	30	1–6
Hereford												
BW	184	1–271	66	1–167	111	1–117	1061	1–14	1204	1–12	171	1–12
WW	168	1–168	61	1–162	53	1–108	978	1–12	1101	1–11	136	1–10
PWG/YW^a^	133	1–133	33	1–125	43	1–114	751	1–11	791	1–11	115	1–10
SDG	110	1–110	33	1–69	59	1–124	366	1–5	762	1–8	127	1–7
SCONF	110	1–110	33	1–69	52	1–117	366	1–5	759	1–8	114	1–7
SFAT	110	1–110	33	1–69	52	1–117	366	1–5	759	1–8	114	1–7

Abbreviations: BW, birth weight; PWG, post‐weaning gain; SCONF, carcass conformation; SDG, slaughter daily gain; SFAT, carcass fatness; WWG, weaning weight gain; YW, yearling weight.

^a^ PWG in Finland and Sweden and YW in Denmark.

### 2.3 Principal Component Analysis and Linkage Disequilibrium Decay

Principal component analyses (PCA) of the genomic data were performed for each breed and country to detect the possible population stratification and similarities between countries for each breed. PCA was done in Plink v 1.7 using the ‐‐PCA command after linkage disequilibrium (LD) pruning by removing SNPs with LD ≥ 0.8 (Purcell et al. 2007).

In order to compare the LD decay pattern for the two breeds in the three different countries, we calculated LD using the ‐‐r2 command in Plink v 1.7 (Purcell et al. 2007). The average LD was calculated as the distance between two adjacent SNPs for every 5 Kb bins using R v 4.1.3 (R Core Team 2022).

### 2.4 Breeding Value Estimation

Pedigree‐based and single‐step genomic breeding values were estimated using multi‐trait animal models in the BLUPf90 and BLUP90iod2 programs, respectively (Misztal et al. 2002; Aguilar et al. 2018). The same statistical model and previously estimated genetic parameters (Table 4) were used in this study as in the recently developed Nordic routine genetic evaluation of beef breeds (Rius‐Vilarrasa et al. 2022). The fixed effects included in the model were country‐sex, country‐twin (twinborn or not), country‐year‐month, country‐dam age‐time, herd‐birth year contemporary group and fixed linear and quadratic regression effects of age at weighing (except for BW) (Table S1). Random effects of animal and residual were included for all traits. For BW, WWG and YW, a random maternal genetic effect and a random permanent environmental effect of the dam were also included in the model. In addition, unknown‐parent groups (UPGs) based on year of birth in 10‐year groups and country of origin (Danish, Finnish, Swedish, European, American, Canadian and other) were included as a random effect. There were 22 UPGs for the Charolais breed and 16 for the Hereford breed. The model was as follows:

$$y=\mathrm{Xb}+Z_{d}u_{d}+Z_{m}u_{m}+\mathrm{Wp}+e$$

where **y** is a vector with observations for the traits, **X**, **Z**_d_, **Z**_m_ and **W** are incident matrices, **b** is a vector of fixed effects, **u**_d_ is a vector of random direct additive genetic effects, **u**_m_ is a vector of random maternal genetic effects for BW, WWG and YW, **p** is a vector of random permanent environmental effects of the dam for BW, WWG and YW (with variance: **I** ⊗ **G**_p_, where **G**_p_ contains the variances and covariances of maternal permanent environmental effects among traits), and **e** is a vector of random residuals (with variance **I** ⊗ **R**_0_, where **R**_0_ contains residual variances and covariances among traits).

**TABLE 4:** Heritability *h* ^2^ and genetic correlations between growth and carcass traits in the Charolais (CHA, below diagonal) and Hereford (HER, above diagonal) breeds, used in the genetic evaluations.

Trait	*h* ^2^_CHA_	*h* ^2^_HER_	Genetic correlations									
			BW‐D	BW‐M	WWG‐D	WWG‐M	YW‐D	YW‐M	PWG	SDG	SCONF	SFAT
BW‐D	0.38	0.47		−0.21	0.43	−0.14	0.53	−0.18	0.34	0.18	0.01	−0.29
BW‐M	0.10	0.11	−0.21		0.12	0.23	0.12	0.35	0.16	0.24	−0.08	0.01
WWG‐D	0.17	0.15	0.35	0.21		−0.16	0.84	−0.13	0.49	0.50	0.10	−0.12
WWG‐M	0.13	0.16	−0.11	0.12	−0.16		−0.07	0.95	−0.02	0.61	0.16	0.29
YW‐D	0.31	0.29	0.48	0.20	0.83	−0.08		−0.15	0.71	0.58	0.07	−0.11
YW‐M	0.10	0.10	−0.17	0.27	−0.09	0.90	−0.15		−0.05	0.60	0.19	0.27
PWG	0.20	0.20	0.31	0.19	0.38	−0.17	0.71	−0.25		0.50	0.03	−0.06
SDG	0.36	0.46	0.17	0.19	0.52	0.54	0.61	0.54	0.40		0.38	0.16
SCONF	0.31	0.28	0.08	0.01	0.07	0.12	0.16	0.14	0.15	0.42		0.11
SFAT	0.34	0.30	−0.23	−0.07	0.00	0.23	−0.05	0.24	−0.02	0.13	−0.10	

Abbreviations: BW, birth weight; D, direct; M, maternal; PWG, post‐weaning gain; SCONF, carcass conformation; SDG, slaughter daily gain; SFAT, carcass fatness; WWG, weaning weight gain; YW, yearling weight.

Assumed distributions for the genetic effects in the pedigree‐based analyses were:

$$\left[\begin{array}{c} u_{d}\\ u_{m} \end{array}\right]\sim N\left(\left[\begin{array}{c} Q_{d}g_{d}\\ Q_{m}g_{m} \end{array}\right],A⨂G_{u}\right)$$

where **Q**_d_ and **Q**_m_ are the incidence matrixes for UPGs **g**_d_ and **g**_m_ are the corresponding random UPG effects (with variance **I** ⊗ **G**_u_), **A** is the numerator relationship matrix and **G**_u_ contains the variances and covariances among (direct and maternal) genetic effects.

In ssGBLUP, the pedigree‐based numerator relationship matrix **A** and the genomic relationship matrix **G** based on genotyped animals were combined to build the relationship matrix **H**. The inverse of the **H** matrix will then be (Misztal et al. 2009):

$$H^{-1}=A^{-1}+\left[\begin{array}{cc} 0 & 0\\ 0 & \;{\left(\alpha G+\left(1-\alpha \right)A_{22}\;\right)}^{-1}-A_{22}^{-1} \end{array}\right]$$

where **G** is the genomic relation matrix for genotyped animals, computed using method 1 by VanRaden (2008), **A**_22_ is the numerator relationship matrix for the genotyped animals, and *α* and 1−α the weights for the **G** and **A** matrix, respectively.

The value of alpha defines the fraction between 0 and 1 of the additive genetic variance that is assumed to be explained by SNP information. For comparison, we used different alpha values (*α*) of 0.95 and 0.70 as weight on genomic information when building the **H** matrix, reflecting that the additive genetic variance is not fully explained by the SNP markers (Goddard et al. 2011).

### 2.5 Genetic Trends

The genetic trends based on EBVs from PBLUP and GEBVs from ssGBLUP using the full data set were compared for each trait and sex‐country‐breed combination, including (G)EBVs for animals born 2010–2021. To facilitate the comparison of trends for EBVs and GEBVs, they were expressed on a similar scale by shifting GEBVs such that the mean GEBV was the same as the mean EBV for animals born in 2010. To be able to compare trends between traits, the mean of (G)EBVs was divided by the additive genetic standard deviation for each trait.

### 2.6 Dispersion and Accuracy Ratio Estimation

The linear regression (LR) method introduced by Legarra and Reverter (2018) was used to estimate bias and accuracy ratios, and relative accuracy improvement. These analyses only included Finnish and Swedish animals, as there were few genotyped Danish animals. Truncated data were generated by removing all phenotypes recorded after 2018 to create a truncated data set. For animals in the validation set (genotyped female animals born after 2018), the LR method was performed to estimate dispersion as *b*1 values and accuracy ratio for each breed and country as described by Legarra and Reverter (2018) using R v4.1.3 (R Core Team 2022). The dispersion (*b*1, here denoted as *b*_*w,p*_) was estimated as the slope of the regression of (G)EBV (*u*) from whole (*w*) data model on (G)EBV from the partial data model (*p*) as follows:

$$b_{w.p}=\frac{\mathrm{cov}\left(u_{w}.u_{p}\right)}{\mathrm{var}\left(u_{p}\right)}$$

The expectation of E(*b*1) is one, and when the *b*1 is less than one it means that (G)EBVs from the partial data set are over dispersed and *vice versa* if it is above one.

The accuracy ratio (*ρ*_*p,w*_) was estimated as the correlation of (G)EBVs (*u*) from partial (*p*) and whole (*w*) model as:

$$\rho_{p.w}=\frac{\mathrm{cov}\left(u_{p.}u_{w}\right)}{\sqrt{\mathrm{var}\left(u_{w}\right)\mathrm{var}\left(u_{p}\right)}}$$

The expectation of E(*ρ*_*p,w*_) ≈ accuracy_*p*_/accuracy_*w*_, that is, an approximation of the ratio between (G)EBV accuracies from the partial and whole model. The lower this accuracy ratio is, the higher the gain is from adding more information in the model.

The dispersion parameter and accuracy ratio were calculated for EBVs from the pedigree‐based BLUP with whole and truncated data, and for GEBVs from ssGBLUP with whole and truncated data using two different alpha values of 0.95 and 0.70. The lm function in R used to estimate dispersion values was also used to obtain their standard errors. Standard errors of accuracy ratios were estimated using bootstrap resampling with 10,000 replicates in the boot package in R (Canty and Ripley 2024).

In addition, the relative improvement in accuracy from switching from the pedigree‐based EBVs to the ssGBLUP GEBVs was calculated using the correlation between EBV and GEBV from the full dataset. We expressed the increase in accuracy relative to the partial evaluation (PBLUP) in percentages (Bonifazi et al. 2022):

$$\mathrm{inc}\_{\mathrm{acc}}_{p,w}=\left(\frac{1}{\rho_{p.w}}-1\right)*100\%$$

## 3 Results

### 3.1 PCA and LD Decay

**FIGURE 1:**
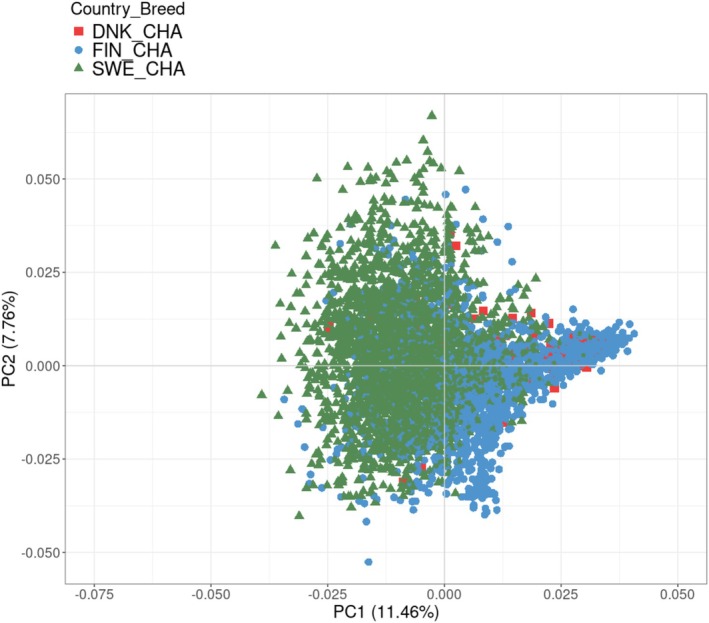
Principal component (PC) analysis of genetic relatedness between Swedish (SWE), Finnish (FIN) and Danish (DNK) Charolais (CHA) cattle. [Colour figure can be viewed at wileyonlinelibrary.com]

**FIGURE 2:**
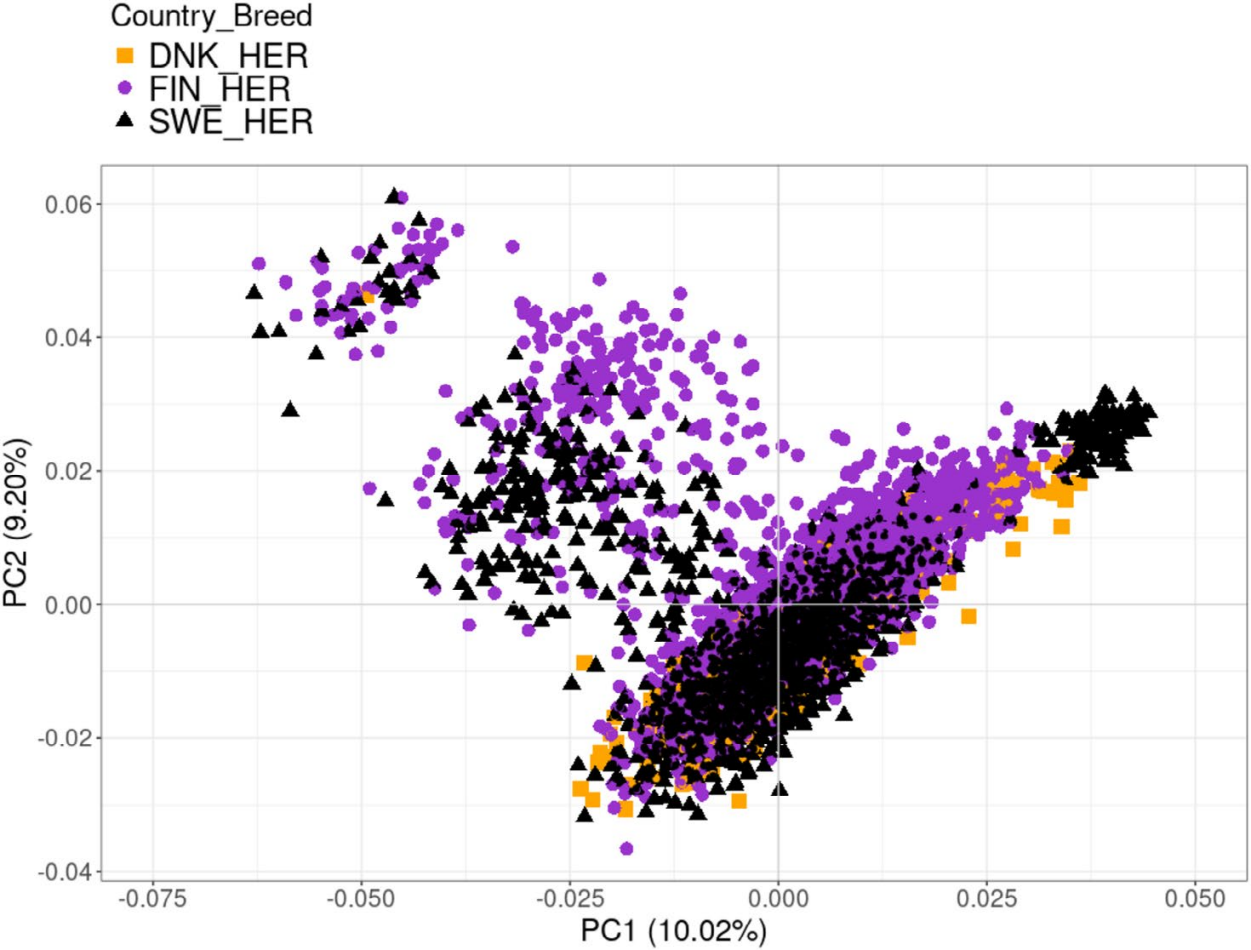
Principal component (PC) analysis of genetic relatedness between Swedish (SWE), Finnish (FIN) and Danish (DNK) Hereford (HER) cattle. [Colour figure can be viewed at wileyonlinelibrary.com]

**FIGURE 3:**
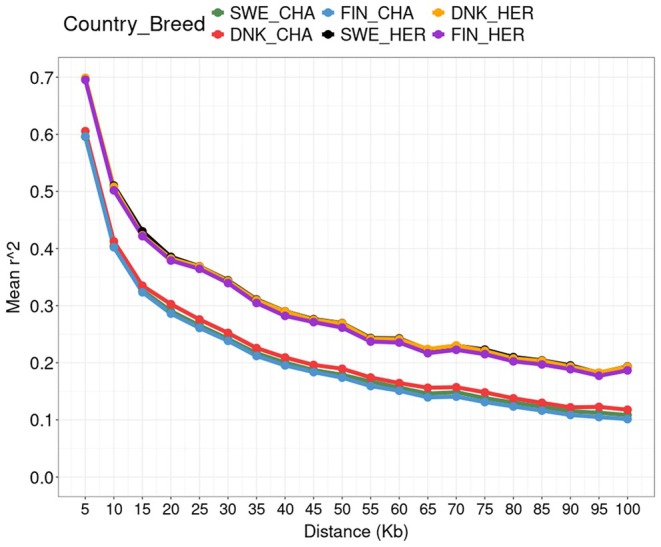
LD decay pattern per breed (CHA = Charolais and HER = Hereford) and country (SWE = Sweden, FIN = Finland and DNK = Denmark). [Colour figure can be viewed at wileyonlinelibrary.com]

The result of the PCA showed no clear stratification between animals from the three different Nordic countries within breed. For Charolais, the first two principal components explained 11.4% and 7.75% of the variance, respectively (Figure 1). The corresponding values were 10.0% for the first and 9.20% for the second principal component in Hereford (Figure 2). The LD decay pattern showed that the Hereford breed had a higher level of LD across distances compared to the Charolais breed (Figure 3). In Hereford, the mean LD (*r* ^2^ value) at the shortest distance (5 Kb) was 0.7, whereas it was 0.6 in Charolais animals (Figure 1).

### 3.2 Genetic Trends

The genetic trends were generally very similar when comparing estimates from PBLUP and ssGBLUP (Table 5). The difference (in genetic standard deviation units) between the mean GEBV and EBV per trait, breed and birth year was at most 0.09 for YW‐D in Danish Charolais born in 2021 (not shown). The largest deviations between trends from PBLUP and ssGBLUP were generally seen for the carcass traits SCONF and SDG, the maternal traits WWG‐M and YW‐M, and also for different traits in the very youngest animals in the data, that is, traits with the lowest reliability of breeding values. However, some of the larger deviations were also seen for WWG‐D in especially Finnish and Danish animals (Table 5).

**TABLE 5:** The mean difference, expressed in genetic standard deviation units, between average GEBV using alpha value of 0.95 and EBV by year, trait and country for Charolais and Hereford cattle.

Trait	Charolais			Hereford		
	SWE	FIN	DNK	SWE	FIN	DNK
BW‐D	0.0000	0.0106	−0.0045	0.0190	0.0192	−0.0096
BW‐M	0.0005	0.0119	−0.0049	0.0003	0.0138	0.0092
WWG‐D	0.0020	0.0417	−0.0104	0.0003	0.0117	0.0225
WWG‐M	−0.0199	0.0221	0.0042	−0.0301	−0.0051	0.0146
YW‐D	−0.0144	0.0281	0.0071	0.0183	0.0108	0.0150
YW‐M	−0.0007	0.0236	−0.0102	−0.0370	−0.0002	0.0131
PWG	−0.0097	−0.0053	0.0097	0.0137	0.0242	−0.0029
SDG	−0.0084	0.0335	−0.0064	−0.0086	0.0188	0.0052
SCONF	−0.0030	0.0150	−0.0026	−0.0453	0.0017	0.0139
SFAT	0.0014	0.0060	0.0010	0.0129	−0.0084	0.0121

Abbreviations: BW, birth weight; D, direct; M, maternal; PWG, post‐weaning gain; SCONF, carcass conformation; SDG, slaughter daily gain; SFAT, carcass fatness; WWG, weaning weight gain; YW, yearling weight.

### 3.3 Dispersion and Accuracy Ratio

For EBVs from PBLUP using the full and truncated data sets, the *b*1 values ranged from as low as 0.64 for SDG in Swedish Charolais animals to 1.10 for SFAT and SCONF in Finnish Charolais animals (Table 6). When comparing GEBVs from ssGBLUP using the full and truncated data sets, the *b*1 values were generally closer to one compared to PBLUP for Charolais. This was also the case for Hereford, except for the maternal genetic traits (Table 7). There was no consistent pattern in dispersion when comparing between the different alpha values. In most cases, the dispersion values were closer to one for Finnish compared to Swedish animals, but not for all traits. For GEBVs, the lowest dispersion value was 0.72 for YW‐M in Swedish Charolais, and the highest value was 1.16 for SCONF in Finnish Hereford animals, both from using an alpha value of 0.70 (Tables 6 and 7).

**TABLE 6:** Dispersion values, with standard errors as subscripts, using the LR method with EBVs from PBLUP and GEBVs from ssGBLUP for growth and carcass traits for female genotyped Charolais animals in the validation set.

Trait	SWE			FIN		
	PBLUP	ssGBLUP *α*_0.70_	ssGBLUP *α*_0.95_	PBLUP	ssGBLUP *α*_0.70_	ssGBLUP *α*_0.95_
BW‐D	0.88_0.05_	0.92_0.04_	0.91_0.04_	0.96_0.05_	0.99_0.05_	0.97_0.04_
BW‐M	0.87_0.04_	0.91_0.04_	0.90_0.04_	0.93_0.04_	0.95_0.03_	0.95_0.03_
WWG‐D	0.73_0.05_	0.86_0.05_	0.86_0.05_	0.92_0.05_	1.00_0.05_	0.99_0.04_
WWG‐M	0.72_0.05_	0.80_0.05_	0.81_0.05_	0.88_0.04_	0.90_0.04_	0.89_0.04_
YW‐D	0.66_0.05_	0.81_0.05_	0.83_0.05_	0.96_0.04_	1.00_0.04_	0.98_0.04_
YW‐M	0.67_0.05_	0.72_0.05_	0.73_0.05_	0.90_0.05_	0.90_0.04_	0.88_0.04_
PWG	0.70_0.04_	0.82_0.04_	0.85_0.04_	1.02_0.03_	0.98_0.03_	0.95_0.03_
SDG	0.64_0.04_	0.76_0.05_	0.77_0.05_	0.94_0.05_	1.02_0.05_	1.01_0.05_
SCONF	0.90_0.03_	0.91_0.03_	0.91_0.03_	1.10_0.03_	1.09_0.03_	1.09_0.03_
SFAT	0.98_0.06_	1.02_0.05_	1.02_0.05_	1.10_0.03_	1.13_0.03_	1.11_0.03_

Abbreviations: BW, birth weight; D, direct; M, maternal; PWG, post‐weaning gain; SCONF, carcass conformation; SDG, slaughter daily gain; SFAT, carcass fatness; WWG, weaning weight gain; YW, yearling weight.

**TABLE 7:** Dispersion values, with standard errors as subscripts, using the LR method with EBVs from PBLUP and GEBVs from ssGBLUP for growth and carcass traits for female genotyped Hereford animals in the validation set.

Trait	SWE			FIN		
	PBLUP	ssGBLUP *α*_0.70_	ssGBLUP *α*_0.95_	PBLUP	ssGBLUP *α*_0.70_	ssGBLUP *α*_095_
BW‐D	0.88_0.06_	0.99_0.04_	0.96_0.04_	0.96_0.05_	1.01_0.04_	0.97_0.03_
BW‐M	0.97_0.04_	1.06_0.04_	1.06_0.04_	1.00_0.04_	1.02_0.03_	1.01_0.03_
WWG‐D	0.85_0.05_	0.98_0.04_	0.96_0.04_	0.71_0.05_	0.92_0.04_	0.93_0.03_
WWG‐M	0.83_0.05_	0.78_0.05_	0.76_0.05_	0.89_0.04_	0.87_0.04_	0.85_0.04_
YW‐D	0.86_0.05_	0.99_0.04_	0.96_0.04_	0.82_0.05_	0.99_0.04_	0.98_0.03_
YW‐M	0.86_0.05_	0.81_0.05_	0.79_0.05_	0.86_0.04_	0.83_0.04_	0.82_0.04_
PWG	0.77_0.05_	0.88_0.04_	0.88_0.04_	0.95_0.04_	1.03_0.04_	1.01_0.03_
SDG	0.75_0.05_	0.81_0.05_	0.79_0.04_	0.79_0.04_	0.91_0.04_	0.93_0.04_
SCONF	1.03_0.05_	1.05_0.04_	1.03_0.04_	1.05_0.04_	1.16_0.03_	1.15_0.03_
SFAT	0.99_0.05_	1.01_0.05_	0.97_0.04_	0.73_0.04_	0.79_0.04_	0.79_0.04_

Abbreviations: BW, birth weight; D, direct; M, maternal; PWG, post‐weaning gain; SCONF, carcass conformation; SDG, slaughter daily gain; SFAT, carcass fatness; WWG, weaning weight gain; YW, yearling weight.

The accuracy ratio for PBLUP was for most traits slightly higher for Finnish compared with Swedish Charolais animals, and ranged from 0.51 for YW‐M in Swedish Charolais to 0.87 for SCONF in Finnish Charolais animals (Table 8). The range of accuracy ratios for ssGBLUP was from 0.55 for YW‐M (alpha value 0.70) in Swedish Charolais to 0.88 for SCONF in Finnish Charolais (for both alpha values) (Table 8). The range of accuracy ratios for PBLUP in Hereford animals was from 0.54 for WWG‐D to 0.77 for BW‐D, both in Finnish animals (Table 9). These values for ssGBLUP ranged between 0.61 for WWG‐M in Swedish and 0.85 for SCONF in Finnish Hereford animals using alpha values of 0.70 and 0.95, respectively (Table 9).

**TABLE 8:** Accuracy ratios, with standard errors as subscripts, using the LR method with EBVs from PBLUP and GEBVs from ssGBLUP, for growth and carcass traits for female genotyped Charolais animals in the validation set.

Trait	SWE			FIN		
	PBLUP	ssGBLUP *α*_0.70_	ssGBLUP *α*_0.95_	PBLUP	ssGBLUP *α*_0.70_	ssGBLUP *α*_0.95_
BW‐D	0.68_0.03_	0.74_0.02_	0.76_0.02_	0.64_0.03_	0.71_0.03_	0.72_0.03_
BW‐M	0.71_0.02_	0.75_0.02_	0.76_0.02_	0.77_0.02_	0.79_0.02_	0.80_0.02_
WWG‐D	0.54_0.04_	0.63_0.03_	0.65_0.03_	0.65_0.03_	0.71_0.03_	0.73_0.03_
WWG‐M	0.57_0.04_	0.60_0.03_	0.62_0.03_	0.69_0.02_	0.71_0.02_	0.72_0.02_
YW‐D	0.54_0.04_	0.62_0.03_	0.65_0.03_	0.72_0.03_	0.76_0.02_	0.77_0.02_
YW‐M	0.51_0.04_	0.55_0.03_	0.56_0.03_	0.67_0.03_	0.70_0.02_	0.70_0.02_
PWG	0.61_0.03_	0.67_0.03_	0.69_0.03_	0.82_0.01_	0.82_0.01_	0.82_0.01_
SDG	0.56_0.04_	0.61_0.03_	0.63_0.03_	0.65_0.03_	0.71_0.02_	0.72_0.02_
SCONF	0.81_0.02_	0.81_0.02_	0.81_0.02_	0.87_0.01_	0.88_0.01_	0.88_0.01_
SFAT	0.63_0.03_	0.69_0.02_	0.71_0.02_	0.84_0.01_	0.85_0.01_	0.85_0.01_

Abbreviations: BW, birth weight; D, direct; M, maternal; PWG, post‐weaning gain; SCONF, carcass conformation; SDG, slaughter daily gain; SFAT, carcass fatness; WWG, weaning weight gain; YW, yearling weight.

**TABLE 9:** Accuracy ratios, with standard errors as subscripts, using the LR method with EBVs from PBLUP and GEBVs from ssGBLUP, for growth and carcass traits for female genotyped Hereford animals in the validation set.

Trait	SWE			FIN		
	PBLUP	ssGBLUP *α*_0.70_	ssGBLUP *α*_0.95_	PBLUP	ssGBLUP *α*_0.70_	ssGBLUP *α*_0.95_
BW‐D	0.59_0.03_	0.74_0.02_	0.77_0.02_	0.62_0.03_	0.75_0.02_	0.77_0.02_
BW‐M	0.74_0.02_	0.78_0.02_	0.79_0.02_	0.77_0.02_	0.81_0.01_	0.81_0.01_
WWG‐D	0.64_0.03_	0.77_0.02_	0.79_0.02_	0.54_0.03_	0.71_0.02_	0.75_0.02_
WWG‐M	0.63_0_._03_	0.61_0.03_	0.62_0.03_	0.71_0.02_	0.69_0.02_	0.68_0.02_
YW‐D	0.61_0.03_	0.76_0.02_	0.79_0.02_	0.58_0.03_	0.73_0.02_	0.76_0.02_
YW‐M	0.66_0.03_	0.63_0.03_	0.64_0.03_	0.68_0.02_	0.66_0.02_	0.66_0.02_
PWG	0.58_0.03_	0.70_0.03_	0.73_0.02_	0.68_0.02_	0.76_0.02_	0.78_0.02_
SDG	0.57_0.03_	0.64_0.03_	0.66_0.03_	0.64_0.03_	0.69_0.02_	0.70_0.02_
SCONF	0.73_0.02_	0.78_0.02_	0.78_0.02_	0.75_0.02_	0.84_0.01_	0.85_0.01_
SFAT	0.67_0.03_	0.72_0.03_	0.73_0.02_	0.64_0.02_	0.67_0.02_	0.68_0.02_

Abbreviations: BW, birth weight; D, direct; M, maternal; PWG, post‐weaning gain; SCONF, carcass conformation; SDG, slaughter daily gain; SFAT, carcass fatness; WWG, weaning weight gain; YW, yearling weight.

### 3.4 Relative Accuracy Improvement

The relative accuracy improvement was in most cases higher for Swedish than for Finnish animals, and tended to be lower for Charolais compared with Hereford animals. It ranged from 3% for SCONF in Finnish Charolais to 30% for SCONF in Swedish Hereford animals using an alpha value of 0.95 (Table 10). Using an alpha value of 0.70, the relative accuracy improvement generally decreased.

**TABLE 10:** Relative accuracy improvement in percentage when shifting from PBLUP to ssGBLUP for female genotyped Charolais and Hereford animals in the validation sets.

Trait	Charolais *α*_0.70_		Charolais *α*_0.95_		Hereford *α*_0.70_		Hereford *α*_0.95_	
	SWE	FIN	SWE	FIN	SWE	FIN	SWE	FIN
BW‐D	5	3	9	6	8	6	13	11
BW‐M	6	10	11	17	12	14	20	22
WWG‐D	7	6	11	10	9	8	16	14
WWG‐M	12	11	20	18	16	16	28	27
YW‐D	6	4	11	8	9	7	15	11
YW‐M	12	10	21	18	15	16	28	29
PWG	5	5	9	8	11	6	19	10
SDG	8	6	14	10	12	8	21	15
SCONF	8	3	15	5	18	8	30	12
SFAT	8	5	15	9	17	12	29	20

Abbreviations: BW, birth weight; D, direct; M, maternal; PWG, post‐weaning gain; SCONF, carcass conformation; SDG, slaughter daily gain; SFAT, carcass fatness; WWG, weaning weight gain; YW, yearling weight.

## 4 Discussion

In this study, we performed single‐step GBLUP for growth and carcass traits in Swedish, Finnish and Danish purebred Charolais and Hereford cattle and estimated different statistics using the LR validation method (Legarra and Reverter 2018). We could show a benefit of using ssGBLUP compared to the conventional PBLUP in the studied beef populations, although the size of the improvements depended on the comparisons made and the alpha value used (0.95 or 0.70).

### 4.1 Genomic Relatedness and LD Pattern Across Countries

The result of PCA showed that the Charolais and Hereford beef breeds across the studied Nordic countries were genomically similar, in that no stratification related to country was detected. This is beneficial for a joint genomic prediction using ssGBLUP in Sweden, Finland and Denmark.

The LD patterns were in agreement with previous studies. For example, a higher LD in Hereford compared to Charolais was shown also by Salomon‐Torres et al. (2014). The higher LD in Hereford may be due to a higher inbreeding level or more intensive selection for economically important traits compared to the Charolais breed (McKay et al. 2007). The LD phase persistency within breed populations is crucial for implementing genomic selection in animal breeding, especially in small‐sized populations (Grossi et al. 2017). Moreover, accuracy in genomic selection depends on the LD between QTLs and markers across the genome (Espigolan et al. 2013; Gurgul et al. 2014). When comparing relative accuracy improvements and prediction accuracy between Charolais and Hereford, the largest improvements were generally seen for Hereford, which could be due to the overall higher LD level in this breed.

In this study, we used the breed code in the ID‐number for animals to divide them into different breeds, and this seems to have worked well for the vast majority of animals. However, there may be a few exceptions for animals that were not completely purebred. This should be revisited before the application of ssGBLUP in the populations. Also, the potential added value of using metafounders (Kudinov et al. 2020; Masuda et al. 2022) could be further investigated in future applications. That would, however, require some additional work with multiple countries involved, such as determining the best number of metafounders in a multi‐population setting and confirming the accuracy of the estimated covariance matrix among metafounders.

### 4.2 Similar Genetic Trends

In general, the genetic trends estimated using ssGBLUP and PBLUP were very similar. In other studies, a divergent point between PBLUP and ssGBLUP occurred at the time when genomic selection was implemented and young animals were selected based on genomic information and Mendelian sampling (Abdollahi‐Arpanahi et al. 2021). However, genomic selection has not yet been in practical use in the Swedish, Finnish and Danish beef cattle populations, and therefore no such difference between the PBLUP and ssGBLUP genetic trends was expected. The largest fluctuation and inconsistencies were observed mainly for maternal traits like WWG‐M and carcass traits like SCONF, for which there was less phenotype information available.

### 4.3 Dispersion and Relative Accuracy Improvement

In the present study, the lowest dispersion values (*b*1) were seen for traits for which the phenotype information for genotyped animals was scarce, such as for YW‐D for which no phenotype information was included for Swedish and Finnish animals, and also SDG for Swedish Charolais cattle when the limited amount of phenotypes was available. In general, the validation based on comparison of EBVs from PBLUP using the full and reduced model gave lower dispersion values that deviated more from one and in most cases less improvement in accuracy as indicated by a lower accuracy ratio, compared to when also genomic information was utilised in ssGBLUP. This is in agreement with previous studies in beef breeds. Adekale et al. (2023) showed that using ssGBLUP could reduce the bias and optimise the dispersion (*b*1) slightly better compared to PBLUP in six German beef breeds. In a study on Hanwoo beef cattle, it was shown that when multi‐trait ssGBLUP was applied instead of PBLUP, the dispersion value became closer to one for carcass weight, but was not changed for the rest of the traits (Mehrban et al. 2019). Bonifazi et al. (2022) found similar or slightly reduced dispersion bias when using ssSNPBLUP compared to PBLUP in the joint evaluation of beef data from several European countries. In simulated beef cattle data, Mouresan et al. (2017) noted improved accuracy when changing from PBLUP to ssGBLUP and the gain in accuracy was largest so when the genotypes of the selection candidates were included in the evaluation.

### 4.4 Effects of Different Alpha Values

Changing (reducing) the alpha value in the blending of the genomic relationship matrix as G_*α*_ = α*G +(1 − *α*)*A_22_ is usually done when the number of genotyped animals is higher than the number of SNPs, in which case creating the inverse of the G matrix can be problematic (VanRaden 2008). This was not the case in our study that comprised a relatively low number of genotyped animals. However, the relative accuracy improvement decreased, and the dispersion value (*b*1) increased to become closer to one for most of the traits when using an alpha value of 0.70 instead of 0.95. The value of alpha defines the proportion of the additive genetic variance that is assumed to be explained by SNP information, and a lower value reflects that the SNP markers do not explain all genetic variation (Goddard et al. 2011). We chose to compare the BLUP90iod2 default alpha value (0.95) with the lower value of 0.7 that had previously been used in a study of single‐step genomic evaluation methods in Finnish beef cattle (Pitkänen et al. 2023), and by NAV for several traits in dairy cattle (Kempe et al. 2024). Assuming a larger residual polygenic proportion (1 − *α*) can be expected to reduce the accuracy of estimated breeding values, particularly for genotyped animals, as more emphasis is put on the less accurate pedigree‐based relationships. The value of alpha has also been reported to influence the inflation of GEBVs, and a lower alpha value was suggested to compensate for the incompleteness of pedigree and incomplete accounting of inbreeding (Misztal et al. 2017). Our results agree with those findings in that dispersion values were generally closer to 1 with a lower alpha value (0.7).

Our findings were in agreement with results by Guarini et al. (2018) who obtained higher reliability of prediction and reduced dispersion when they used an alpha value of 0.80 instead of 0.95 in Holstein cattle. An alpha value as low as 0.50 was used for ssGBLUP in Australian sheep breeds by McMillan and Swan (2017). They showed that when the alpha value increased, the dispersion increased, although it differed among traits, and that the accuracy of GEBVs increased with higher alpha values (McMillan and Swan 2017). However, the same authors suggested that a wide range of alpha values can be used as the loss of accuracy was minimal and no impact on the ranking of animals was seen (McMillan and Swan 2017). In Australian beef cattle, Zhang et al. (2017) showed, similarly to the present study, that when the alpha value increased, the accuracy of ssGBLUP increased. They suggested an optimal range of alpha value from 0.4 to 0.8 in the presence of adequate phenotypes.

### 4.5 Choice of Validation Method

Cross‐validation is often regarded as the gold standard method for validation, but there are situations when it is difficult to apply (Runcie and Cheng 2019). This is, for example, the case when predicting maternal effects for which there are no direct observations, and when progeny group sizes are small; in such cases, cross‐validation can lead to contradictory results (Legarra and Reverter 2018). In our study, cross‐validation was not a reliable method due to relatively few animals with both phenotype and genotype information in the validation set, especially for carcass traits (*N* < 30). It is likely that with a higher number of genotyped animals with phenotype records, both in the reference and (especially) in the validation set, cross‐validation would have been an option to use, at least for some of the traits. The number of genotyped animals in the beef recording schemes is expected to increase with time, which will benefit practical applications in the Nordic countries. Lourenco et al. (2015) reported higher predictivity when using a larger reference population for ssGBLUP for growth traits in Angus. Improvements in prediction accuracy when using ssGBLUP were also reported for yearling weight and carcass weight in Hanwoo cattle (Mehrban et al. 2019).

For the maternal components, difficulties to use cross‐validation would still remain; however, Romé et al. (2023) got contradicting results when comparing models with and without covariance between the direct and maternal genetic effects when using forward cross‐validation and the LR method. Their analysis of simulated data including maternal genetic effects showed that cross‐validation, commonly done in animal breeding, led to biased ranking of models based on prediction accuracy. For this reason, we instead chose to use the LR method based on (G)EBVs in this study (Legarra and Reverter 2018). Maternal traits are especially complex in that they are first measured when the next generation is born, and maternal and direct genetic effects tend to be correlated. Maternal traits benefit from including genomic information, and Lourenco et al. (2013) showed in simulation studies that the prediction accuracy for maternal effects can be as high as direct effects when using ssGBLUP. A larger increase in population accuracy for maternal than for direct breeding values for weaning weight in Limousine was seen by Bonifazi et al. (2022) when moving from PBLUP to ssSNPBLUP.

## 5 Conclusion

We conclude that introducing single‐step GBLUP evaluation of beef breeds jointly in Sweden, Finland and Denmark would be feasible and beneficial in that it would improve breeding value accuracy, especially in young genotyped animals, as well as dispersion (*b*1 values), compared to the current conventional PBLUP evaluation. The choice of the weight put on genomic information, the alpha value, had some influence on the validation results. Lowering the alpha value from 0.95 to 0.70 generally improved the dispersion values but reduced both the accuracy ratio (using the LR method) and relative accuracy improvement by shifting from PBLUP to GBLUP for most of the traits.

## Author Contributions

S. Eriksson and F. Fikse designed and supervised the study. A. Nazari‐Ghadikolaei carried out the data analysis with contributions from F. Fikse. A. Nazari‐Ghadikolaei and S. Eriksson drafted the manuscript with contributions from F. Fikse. All authors read, edited and approved the final manuscript.

## Ethics Statement

DNA samples used for genotyping were collected within this research project with an ethical permit (Dnr 5.8.18‐05179/2019) issued by the Swedish Board of Agriculture. The rest of the genotypes analyzed in this study were from samples previously collected by parties outside this project.

## Conflicts of Interest

The Nordic Cattle Genetic Evaluation has provided the data for this study and Freddy Fikse was employed by one of the owner organisations, Växa. We declare that there are no other conflicts of interest.

## Supporting information


**Table S1:** Traits and number of levels of fixed class effects for growth and carcass traits for Charolais and Hereford animals.

## Data Availability

Restrictions apply to the availability of these data, which were used under licence for this study. Data are available from the authors with the permission of VikingGenetics and the Nordic Cattle Genetic Evaluation with its owner organisations.

## References

[jbg70018-bib-0001] Abdollahi‐Arpanahi, R. , D. Lourenco , and I. Misztal . 2021. “Detecting Effective Starting Point of Genomic Selection by Divergent Trends From BLUP and ssGBLUP in Pigs, Beef Cattle, and Broilers.” Journal of Animal Science 99: skab243. 10.1093/jas/skab243. 34390341 PMC8420679

[jbg70018-bib-0002] Adekale, D. , H. Alkhoder , Z. Liu , D. Segelke , and J. Tetens . 2023. “Single‐Step SNPBLUP Evaluation in Six German Beef Cattle Breeds.” Journal of Animal Breeding and Genetics 140: 496–507. 10.1111/jbg.12774. 37061869

[jbg70018-bib-0003] Aguilar, I. , S. Tsuruta , Y. Masuda , D. Lourenco , A. Legarra , and I. Misztal . 2018. “BLUPF90 Suite of Programs for Animal Breeding With Focus on Genomics.” In *Proceedings of the World Congress on Genetics Applied to Livestock Production*, 11–16.

[jbg70018-bib-0004] Bonifazi, R. , M. P. L. Calus , J. ten Napel , et al. 2022. “International Single‐Step SNPBLUP Beef Cattle Evaluations for Limousin Weaning Weight.” Genetics, Selection, Evolution: GSE 54: 57. 10.1186/s12711-022-00748-0. 36057564 PMC9441073

[jbg70018-bib-0005] Canty, A. , and B. D. Ripley . 2024. “Boot: Bootstrap R (S‐Plus) Functions.” R Package (Version 1.3‐3.1).

[jbg70018-bib-0006] Clasen, J. B. , C. Bengtsson , H. N. Källström , E. Strandberg , W. F. Fikse , and L. Rydhmer . 2021. “Dairy Cattle Farmers' Preferences for Different Breeding Tools.” Animal 15, no. 12: 100409. https://www.sciencedirect.com/science/article/pii/S1751731121002524?via%3Dihub. 34839224 10.1016/j.animal.2021.100409

[jbg70018-bib-0007] Espigolan, R. , F. Baldi , A. A. Boligon , et al. 2013. “Study of Whole Genome Linkage Disequilibrium in Nellore Cattle.” BMC Genomics 14: 1–8. 10.1186/1471-2164-14-305. 23642139 PMC3662636

[jbg70018-bib-0008] Goddard, M. E. , B. J. Hayes , and T. H. Meuwissen . 2011. “Using the Genomic Relationship Matrix to Predict the Accuracy of Genomic Selection.” Journal of Animal Breeding and Genetics 128, no. 6: 409–421. 10.1111/j.1439-0388.2011.00964.x. 22059574

[jbg70018-bib-0009] Grossi, D. A. , M. Jafarikia , L. F. Brito , M. E. Buzanskas , M. Sargolzaei , and F. S. Schenkel . 2017. “Genetic Diversity, Extent of Linkage Disequilibrium and Persistence of Gametic Phase in Canadian Pigs.” BMC Genetics 18, no. 1: 1–13. 10.1186/s12863-017-0473-y. 28109261 PMC5251314

[jbg70018-bib-0010] Guarini, A. , D. Lourenco , L. Brito , et al. 2018. “Comparison of Genomic Predictions for Lowly Heritable Traits Using Multi‐Step and Single‐Step Genomic Best Linear Unbiased Predictor in Holstein Cattle.” Journal of Dairy Science 101, no. 9: 8076–8086. 10.3168/jds.2017-14193. 29935829

[jbg70018-bib-0011] Guinan, F. L. , G. R. Wiggans , H. D. Norman , et al. 2023. “Changes in Genetic Trends in US Dairy Cattle Since the Implementation of Genomic Selection.” Journal of Dairy Science 196, no. 2: 1110–1129. 10.3168/jds.2022-22205. 36494224

[jbg70018-bib-0012] Gurgul, A. , E. Semik , K. Pawlina , T. Szmatoła , I. Jasielczuk , and M. Bugno‐Poniewierska . 2014. “The Application of Genome‐Wide SNP Genotyping Methods in Studies on Livestock Genomes.” Journal of Applied Genetics 55: 197–208. 10.1007/s13353-014-0202-4. 24566962

[jbg70018-bib-0013] Hietala, S. , A.‐M. Leino , J. Nousiainen , and A. Huuskonen . 2022. “Improving Efficiency of Finnish Beef Production Through Breeding With Genomic Selection Effects Climate Impact of Beef.” In *Proceedings of 13th International Conference on Life Cycle Assessment of Food* (LCA Foods 2022), 12–14 October 2022, Lima, Peru. PELCAN‐PUCP, Lima, Peru.

[jbg70018-bib-0014] Kempe, R. , M. Koivula , T. J. Pitkänen , et al. 2024. “Single‐Step Genomic Prediction Models for Metabolic Body Weight in Nordic Holstein, Red Dairy Cattle, and Jersey.” Interbull Bulletin 60: 92–96.

[jbg70018-bib-0015] Kudinov, A. , E. Mäntysaari , G. Aamand , P. Uimari , and I. Strandén . 2020. “Metafounder Approach for Single‐Step Genomic Evaluations of Red Dairy Cattle.” Journal of Dairy Science 103, no. 7: 6299–6310. 10.3168/jds.2019-17483. 32418688

[jbg70018-bib-0016] Legarra, A. , O. F. Christensen , I. Aguilar , and I. Misztal . 2014. “Single Step, a General Approach for Genomic Selection.” Livestock Science 166: 54–65. 10.1016/j.livsci.2014.04.029.

[jbg70018-bib-0017] Legarra, A. , and A. Reverter . 2018. “Semi‐Parametric Estimates of Population Accuracy and Bias of Predictions of Breeding Values and Future Phenotypes Using the LR Method.” Genetics, Selection, Evolution 50: 1–18. 10.1186/s12711-018-0426-6. PMC6219059 30400768

[jbg70018-bib-0018] Lourenco, D. , I. Misztal , H. Wang , I. Aguilar , S. Tsuruta , and J. Bertrand . 2013. “Prediction Accuracy for a Simulated Maternally Affected Trait of Beef Cattle Using Different Genomic Evaluation Models.” Journal of Animal Science 91, no. 9: 4090–4098. 10.2527/jas.2012-5826. 23893997

[jbg70018-bib-0019] Lourenco, D. , S. Tsuruta , B. Fragomeni , et al. 2015. “Genetic Evaluation Using Single‐Step Genomic Best Linear Unbiased Predictor in American Angus.” Journal of Animal Science 93, no. 6: 2653–2662. 10.2527/jas.2014-8836. 26115253

[jbg70018-bib-0020] Masuda, Y. , P. M. VanRaden , S. Tsuruta , D. A. Lourenco , and I. Misztal . 2022. “Invited Review: Unknown‐Parent Groups and Metafounders in Single‐Step Genomic BLUP.” Journal of Dairy Science 105, no. 2: 923–939. 10.3168/jds.2021-20293. 34799109

[jbg70018-bib-0021] McKay, S. D. , R. D. Schnabel , B. M. Murdoch , et al. 2007. “Whole Genome Linkage Disequilibrium Maps in Cattle.” BMC Genetics 8, no. 1: 1–12. 10.1186/1471-2156-8-74. 17961247 PMC2174945

[jbg70018-bib-0022] McMillan, A. , and A. Swan . 2017. “Weighting of Genomic and Pedigree Relationships in Single Step Evaluation of Carcass Traits in Australian Sheep.” Proceedings of the Association for the Advancement of Animal Breeding and Genetics 22: 557–560.

[jbg70018-bib-0023] Mehrban, H. , D. H. Lee , M. Naserkheil , M. H. Moradi , and N. Ibáñez‐Escriche . 2019. “Comparison of Conventional BLUP and Single‐Step Genomic BLUP Evaluations for Yearling Weight and Carcass Traits in Hanwoo Beef Cattle Using Single Trait and Multi‐Trait Models.” PLoS One 14, no. 10: e0223352. 10.1371/journal.pone.0223352. 31609979 PMC6791548

[jbg70018-bib-0024] Meuwissen, T. , B. Hayes , and M. Goddard . 2016. “Genomic Selection: A Paradigm Shift in Animal Breeding.” Animal Frontiers 6, no. 1: 6–14. 10.2527/af.2016-0002.

[jbg70018-bib-0025] Misztal, I. , H. L. Bradford , D. A. L. Lourenco , et al. 2017. “Studies on Inflation of GEBV in Single‐Step GBLUP for Type.” Interbull Bulletin 51: 38–42.

[jbg70018-bib-0026] Misztal, I. , A. Legarra , and I. Aguilar . 2009. “Computing Procedures for Genetic Evaluation Including Phenotypic, Full Pedigree, and Genomic Information.” Journal of Dairy Science 92, no. 9: 4648–4655. 10.3168/jds.2009-2064. 19700728

[jbg70018-bib-0027] Misztal, I. , S. Tsuruta , T. Strabel , B. Auvray , T. Druet , and D. Lee . 2002. “BLUPF90 and Related Programs (BGF90).” In *Proceedings of the 7th World Congress on Genetics Applied to Livestock Production*.

[jbg70018-bib-0028] Mouresan, E. F. , J. Altarriba , C. Moreno , S. Munilla , A. González‐Rodríguez , and L. Varona . 2017. “Performance of Genomic Selection Under a Single‐Step Approach in Autochthonous Spanish Beef Cattle Populations.” Journal of Animal Breeding and Genetics 134, no. 4: 289–299. 10.1111/jbg.12253. 28164382

[jbg70018-bib-0029] NAV . 2024. “Beef Publication.” https://nordicebv.info/beef‐cattle/beef‐publication/.

[jbg70018-bib-0030] Pitkänen, T. , A.‐M. Leino , M. Taskinen , E. Mäntysaari , and I. Strandén . 2023. “Multibreed Single‐Step Genomic Evaluation Model for Finnish Beef Cattle.” https://interbull.org/static/web/4_Pitkanen_Interbull2023_Pitkanen.pdf.

[jbg70018-bib-0031] Purcell, S. , B. Neale , K. Todd‐Brown , et al. 2007. “PLINK: A Tool Set for Whole‐Genome Association and Population‐Based Linkage Analyses.” American Journal of Human Genetics 81, no. 3: 559–575. 10.1086/519795. 17701901 PMC1950838

[jbg70018-bib-0032] R Core Team . 2022. R: A Language and Environment for Statistical Computing. R Foundation for Statistical Computing. https://www.r‐project.org/.

[jbg70018-bib-0033] Rius‐Vilarrasa, E. , F. Fikse , J. Pöso , K. Byskov , and G. Aamand . 2022. “Nordic Genetic Evaluation for Purebred Beef Cattle.” https://interbull.org/static/web/220530_15h00_MONTREAL4‐5_ElisendaRius‐Vilarrasa.pdf.

[jbg70018-bib-0034] Romé, H. , T. T. Chu , D. Marois , C.‐H. Huang , P. Madsen , and J. Jensen . 2023. “Estimation and Consequences of Direct‐Maternal Genetic and Environmental Covariances in Models for Genetic Evaluation in Broilers.” Genetics Selection Evolution 55: 58. 10.1186/s12711-023-00829-8. PMC10405509 37550635

[jbg70018-bib-0035] Runcie, D. , and H. Cheng . 2019. “Pitfalls and Remedies for Cross Validation With Multi‐Trait Genomic Prediction Methods.” G3 9, no. 11: 3727–3741. 10.1534/g3.119.400598. 31511297 PMC6829121

[jbg70018-bib-0036] Saatchi, M. , J. Ward , and D. Garrick . 2013. “Accuracies of Direct Genomic Breeding Values in Hereford Beef Cattle Using National or International Training Populations.” Journal of Animal Science 91, no. 4: 1538–1551. 10.2527/jas.2012-5593. 23345550

[jbg70018-bib-0037] Salomon‐Torres, R. , L. K. Matukumalli , C. P. Van Tassell , C. Villa‐Angulo , V. M. Gonzalez‐Vizcarra , and R. Villa‐Angulo . 2014. “High Density LD‐Based Structural Variations Analysis in Cattle Genome.” PLoS One 9, no. 7: e103046. 10.1371/journal.pone.0103046. 25050984 PMC4106904

[jbg70018-bib-0038] Terry, S. A. , J. A. Basarab , L. L. Guan , and T. A. McAllister . 2021. “Strategies to Improve the Efficiency of Beef Cattle Production.” Canadian Journal of Animal Science 101, no. 1: 1–19. 10.1139/cjas-2020-0022.

[jbg70018-bib-0039] VanRaden, P. M. 2008. “Efficient Methods to Compute Genomic Predictions.” Journal of Dairy Science 91, no. 11: 4414–4423. 10.3168/jds.2007-0980. 18946147

[jbg70018-bib-0040] Zhang, Y. , A. Swan , D. Johnston , and C. Girard . 2017. “Weighting Factors for Genomic Information Used in Single‐Step Genomic Selection in Australian Beef.” Proceedings of the Association for the Advancement of Animal Breeding and Genetics 22: 309–312.

