# Supplementary material for: Single‐Step Genomic Predictions for Growth and Carcass Traits in Nordic Charolais and Hereford Cattle
Source: J Anim Breed Genet. 2025 Oct 2;143(2):283–95. doi: 10.1111/jbg.70018 (PMC12887150; doi:10.1111/jbg.70018)
Supplement: Supplementary file 1 — Table S1: Traits and number of levels of fixed class effects for growth and carcass traits for Charolais and Hereford animals. [file JBG-143-283-s001.docx]

**Supplementary Table 1.** Traits and number of levels of fixed class effects for growth and carcass traits for Charolais and Hereford animals.

|  |  | **N levels of fixed effects** | |
| --- | --- | --- | --- |
| **Trait^1^** | **Fixed Effect** | **Charolais** | **Hereford** |
| BW | Year*month | 1236 | 1170 |
| WWG | Year*month | 888 | 864 |
| PWG | Year*month | 514 | 431 |
| YW | Year*month | 305 | 395 |
| SDG, SCONF, SFAT | Year*month | 795 | 756 |
| BW | Country*Sex | 6 | 6 |
| WWG | Country*Sex | 6 | 6 |
| PWG | Country*Sex | 4 | 4 |
| YW | Country*Sex | 2 | 2 |
| SDG, SCONF, SFAT | Country*Sex | 6 | 6 |
| BW | Country*Twin | 6 | 6 |
| WWG | Country*Twin | 6 | 6 |
| PWG | Country*Twin | 4 | 4 |
| YW | Country*Twin | 2 | 2 |
| SDG, SCONF, SFAT | Country*Twin | 6 | 6 |
| BW | Country*dam_age*sex | 111 | 111 |
| WWG | Country*dam_age*sex | 111 | 111 |
| PWG | Country*dam_age*sex | 74 | 74 |
| YW | Country*dam_age*sex | 37 | 37 |
| SDG, SCONF, SFAT | Country*dam_age*sex | 99 | 99 |

^1^BW = birth weight; WWG = weaning weight gain; PWG = post-weaning gain; YW = yearling weight; SDG = slaughter daily gain; SCONF = carcass conformation; SFAT = carcass fatness.
